# CXCL12 expression by healthy and malignant ovarian epithelial cells

**DOI:** 10.1186/1471-2407-11-97

**Published:** 2011-03-16

**Authors:** Véronique Machelon, Françoise Gaudin, Sophie Camilleri-Broët, Salam Nasreddine, Laurence Bouchet-Delbos, Eric Pujade-Lauraine, Jerôme Alexandre, Laurence Gladieff, Fernando Arenzana-Seisdedos, Dominique Emilie, Sophie Prévot, Philippe Broët, Karl Balabanian

**Affiliations:** 1 INSERM UMR_S 996, Université Paris-Sud 11, Clamart, 92140 France; 2 JE2492, Assistance-Publique Hôpitaux de Paris (AP-HP), Université Paris-Sud 11, Villejuif, 94800 France; 3 Cabinet de Pathologie Tolbiac, Paris, 75013 France; 4 Service d'Oncologie médicale, AP-HP, Université Paris Descartes, Hôpital Hôtel-Dieu, Paris, 75001 France; 5 Centre Claudius Regaud, Toulouse, 33000 France; 6 INSERM U819, Laboratoire de Pathogénie Virale, Institut Pasteur, Paris, 75015 France; 7 Service de Microbiologie-Immunologie Biologique, AP-HP, Université Paris-Sud 11, Hôpital Antoine-Béclère, Clamart, 92140 France; 8 Service d'Anatomie et de Cytologie Pathologiques, AP-HP, Université Paris-Sud 11, Hôpital Antoine-Béclère, Clamart, 92140 France

## Abstract

**Background:**

CXCL12 has been widely reported to play a biologically relevant role in tumor growth and spread. In epithelial ovarian cancer (EOC), CXCL12 enhances tumor angiogenesis and contributes to the immunosuppressive network. However, its prognostic significance remains unclear. We thus compared CXCL12 status in healthy and malignant ovaries, to assess its prognostic value.

**Methods:**

Immunohistochemistry was used to analyze CXCL12 expression in the reproductive tracts, including the ovaries and fallopian tubes, of healthy women, in benign and borderline epithelial tumors, and in a series of 183 tumor specimens from patients with advanced primary EOC enrolled in a multicenter prospective clinical trial of paclitaxel/carboplatin/gemcitabine-based chemotherapy (GINECO study). Univariate COX model analysis was performed to assess the prognostic value of clinical and biological variables. Kaplan-Meier methods were used to generate progression-free and overall survival curves.

**Results:**

Epithelial cells from the surface of the ovary and the fallopian tubes stained positive for CXCL12, whereas the follicles within the ovary did not. Epithelial cells in benign, borderline and malignant tumors also expressed CXCL12. In EOC specimens, CXCL12 immunoreactivity was observed mostly in epithelial tumor cells. The intensity of the signal obtained ranged from strong in 86 cases (47%) to absent in 18 cases (<10%). This uneven distribution of CXCL12 did not reflect the morphological heterogeneity of EOC. CXCL12 expression levels were not correlated with any of the clinical parameters currently used to determine EOC prognosis or with HER2 status. They also had no impact on progression-free or overall survival.

**Conclusion:**

Our findings highlight the previously unappreciated constitutive expression of CXCL12 on healthy epithelia of the ovary surface and fallopian tubes, indicating that EOC may originate from either of these epithelia. We reveal that CXCL12 production by malignant epithelial cells precedes tumorigenesis and we confirm in a large cohort of patients with advanced EOC that CXCL12 expression level in EOC is not a valuable prognostic factor in itself.

**Trial Registration:**

ClinicalTrials.gov: NCT00052468

## Background

Epithelial ovarian cancer (EOC) has one of the highest mortality rates of all gynecologic malignancies. It is the sixth most common cancer and the fifth most common cause of cancer-related death among women in developed countries [1]. Due to the silent nature of early-stage disease, most women with EOC have disseminated disease (*i.e. *expansion in the peritoneum and metastasis in the omentum) at the time of diagnosis and present an advanced stage of the disease, with a five-year survival rate below 30% [2]. Despite the high incidence and mortality rates, the etiology of EOC and the molecular pathways underlying its progression remain poorly understood. According to the International Federation of Gynecology and Obstetrics (FIGO), clinical stage, histologic grade and postoperative residual tumor mass are the most important prognostic factors in patients with EOC [3]. However, clinical factors and derivative prognostic models remain inadequate for the accurate prediction of outcome for a specific patient, indicating a need for the identification of biological factors to improve prognostic assessment. This aspect has recently been addressed with the identification of several biomarkers for the identification of histologic subtypes and the more accurate prediction of patient outcome [4-9]. Chemokines and their receptors have been known for many years to influence the development of primary epithelial tumors, in which they regulate the proliferation and survival of tumor cells, tumor-infiltrating leukocytes, angiogenesis and metastasis [10-12]. In epithelial cancers, these molecules play a key role in controlling both autocrine and paracrine communication between the different cell types of the tumor microenvironment [13]. Thus, chemokines and their receptors may constitute new biomarkers of potential prognostic value in various cancers, including EOC.

In this study, we focused on the α-chemokine stromal cell-derived factor-1 (SDF-1)/CXCL12, which, together with its receptors CXCR4 and CXCR7, constitutes the chemokine/receptor axis attracting the greatest level of interest in oncology [11,14]. In EOC, CXCL12 products (*i.e. *protein and mRNA) have been detected in tumor cells [15,16]. We previously showed that CXCL12 orchestrates the recruitment of pre-DC2s and protects them from tumor macrophage IL-10-promoted apoptosis, thereby contributing to the immunosuppressive network within the tumor microenvironment [15]. In addition, CXCL12 regulates tumor angiogenesis, a critical step in tumor growth. Indeed, we have shown that hypoxia triggers the production of CXCL12 and vascular endothelium growth factor (VEGF) by EOC, with these two molecules acting in synergy to enhance tumor angiogenesis *in vivo *[17]. CXCL12 also acts on tumor cell proliferation and survival and, through its main receptor CXCR4, governs the migration of malignant cells and their invasion of the peritoneum, a major route for ovarian cancer spread [16,18-20]. Other factors must also been considered, but previous observations strongly suggest that CXCL12 provides the autocrine and paracrine signals controlling malignant progression in EOC [11]. Some recent studies have investigated CXCL12 status in EOC, and reported no prognostic significance of CXCL12 production [11,21,22]. However, the results were obtained with ovarian cancer specimens from patients undergoing chemotherapy via heterogeneous protocols, with a follow-up period of less than four years. The prognostic significance of CXCL12 production by ovarian cancer cells remains to be clearly assessed in larger cohorts of EOC patients undergoing the same type of chemotherapy and followed up for longer periods. Furthermore, the pattern of CXCL12 expression in healthy ovaries and in benign and borderline ovarian tumors has scarcely been investigated. Elucidation of these points is required to determine whether CXCL12 production is associated with the malignant process and whether it constitutes a valuable prognostic factor in EOC.

In this study, we investigated CXCL12 status in the reproductive tracts of healthy women. We studied the ovarian surface epithelium (OSE) and fallopian tubes, both of which are considered probable sources of EOC [23,24]. We also investigated CXCL12 status in benign and borderline epithelial tumors, and in a series of 183 patients with advanced primary EOC enrolled in a multicenter prospective clinical trial of paclitaxel/carboplatin/gemcitabine (TCG)-based chemotherapy [ClinicalTrials.gov Identifier: NCT00052468]. We quantified CXCL12 by immunohistochemistry (IHC) in EOC specimens and further assessed its potential association with clinical and pathologic features, including staging parameters and tumor histotypes, and with the expression of HER2, a tyrosine kinase receptor that may influence outcome when overexpressed [21,25]. Finally, we investigated whether the production of CXCL12 within the tumor affected progression-free survival (PFS) and the overall survival (OS) of patients with advanced primary EOC.

## Methods

### Ethics statement

We included 183 patients with advanced primary EOC (FIGO stage Ic-IV) in this study. All had been enrolled in the GERCOR-AGO-OVAR-9 large phase III randomized trial of first-line TCG-based chemotherapy (GINECO study) conducted at 58 French centers from July 2002 to April 2004 [ClinicalTrials.gov Identifier: NCT00052468] [25-27]. Formalin-fixed, paraffin-embedded tumors from primary surgery were obtained with the approval of the institutional review board of the corresponding center (CCPPRB number: 02780) after inclusion of the patient in the clinical trial. Formalin-fixed and paraffin-embedded specimens recovered from five healthy ovaries (mostly contralateral to the malignant ovary), eight benign tumors (4 serous and 4 mucinous), eight borderline tumors (4 serous and 4 mucinous), and three non epithelial ovarian tumors (2 granulosa tumors and 1 dysgerminoma) were provided from the archives of patients treated at Antoine-Béclère Hospital (Service d'Anatomie et de Cytologie Pathologiques, Clamart, France) between 1998 and 2007. Approval was obtained from the ethics commission of Antoine-Béclère Hospital for all analyses of tumor material from the archives initially obtained for routine diagnostic and therapeutic purposes. This study was carried out in accordance with good clinical practice guidelines, national laws, and the Declaration of Helsinki. All patients provided written informed consent.

### Cell enrichment

Tumor cell enrichment from malignant ascites was based on the expression of CD326, a human epithelial antigen also known as EpCAM, one of the most frequently identified and highly expressed biomarkers in EOC [28]. CD326^+ ^cells were positively selected on AutoMACs columns (Myltenyi Biotech, Paris, France), from ascites samples collected with ethics committee (Antoine-Béclère Hospital) approval from one patient (FIGO stage IV) diagnosed with invasive EOC with peritoneal extension, as previously described [29]. In the positive fraction, the percentage of CD326^+ ^cells was >80%, whereas the negative fraction contained mostly CD45^+ ^leukocytes, as determined by flow cytometry (FACSCalibur, BD Biosciences, Le Pont De Claix, France) with FITC-conjugated anti-human CD45 (clone H130, IgG1, BD Biosciences) and PE-conjugated anti-human CD326 (clone HEA 125, IgG1, Myltenyi Biotec) monoclonal antibodies (mAb). Ascites samples were also analyzed for CXCL12 content with the human CXCL12/SDF-1α Quantikine ELISA kit (R&D Systems, Lille, France), according to the manufacturer's instructions.

### Immunostaining grading and score

CXCL12 was localized immunohistochemically on 4 μm sections of paraffin-embedded tissues (healthy ovaries, benign, borderline and non epithelial tumors) and on tissue microarrays (TMAs) of EOC specimens. Identical experimental protocols were used for immunohistochemistry (IHC) on conventional slides and TMAs. Sections were deparaffinized and rehydrated and then treated with citrate buffer pH6 and heated in a microwave oven. For CXCL12 immunostaining, we used a mAb against CXCL12 (clone K15C, IgG2a) at a concentration of 1.37 μg/ml. This mAb has already been widely used for the detection of CXCL12 in mesothelial cells, ovarian cancer cells and breast carcinomas [15,30-33]. The binding of the K15C mAb was detected by the streptavidine-biotin peroxidase method (LSAB kit, Dako, Trappes, France). Sections were then counterstained with hematoxylin. Images were obtained with a Leica DMLB microscope equipped with standard optic objectives, at the indicated magnifications, and digitized directly with a Sony 3CCD color video camera.

Immunostaining for CXCL12 was then scored by two independent investigators (F.G. and S.C-B), as follows: an intensity score of 0 if negative, 1 (weak intensity), 2 (moderate intensity) or 3 (strong intensity), added to a score for the percentage of positive cells, of 0 (0%), 1 (1-10%), 2 (10-50%), 3 (50-80%) or 4 (>80%), as recently reported [29]. Tumor specimens scoring 0 to 4 were considered to display low-moderate levels of CXCL12 expression (CXCL12^low/moderate^, n = 97), whereas those scoring 5 to 7 were classified as having high levels of CXCL12 expression (CXCL12^high^, n = 86). For the 183 patients included in the GINECO clinical trial, results were compared with IHC for HER2, as previously described [26].

### RT-PCR analyses

Total cellular RNA was extracted from freshly frozen ovarian tissue samples, with the RNeasy Mini kit (Qiagen, Courtaboeuf, France). It was then reverse-transcribed with random hexamers (Roche Diagnostics, Meylan, France) and Moloney murine leukemia virus reverse transcriptase (Fisher Bioblock, Illkirch, France). The resulting cDNAs (1 μg) were then amplified by semi-quantitative PCR (2 min at 94°C followed by 33 cycles of 30 s at 61°C or 55°C for *CXCL12 *and *β-actin*, respectively) with forward (658-677) 5'-GGGCTCCTGGGTTTTGTATT-3' and reverse (1056-1075) 5'-GTCCTGAGAGTCCTTTTGCG-3' primers for *CXCL12 *(417 bp), and forward (214-223) 5'-GGGTCAGAAGGATTCCTATG-3' and reverse (432-451) 5'-GGTCTCAAACATGATCTGGG-3' primers for *β-actin *(237 bp). Quantitative real-time PCR was performed on a Light Cycler instrument (LC480, Roche Diagnostics) with the LightCycler 480 SYBR Green detection kit (Roche Diagnostics) and forward (178-203) 5'-GTCAAGCATCTCAAAATTCTCAACAC-3' and reverse (262-281) 5'-CACTTTAGCTTCGGGTCAATGC-3' primers for *CXCL12 *(103 bp), and forward (214-223) 5'-GGGTCAGAAGGATTCCTATG-3' and reverse (432-451) 5'-GGTCTCAAACATGATCTGGG-3' primers for *β-actin *(237 bp). We used the ABI 7300 Sequence Detection System (Applied Biosystems, Courtaboeuf, France) with the following amplification scheme: 95°C for 10 min and 45 cycles of 95°C for 10 s, 68°C for 10 s and 72°C for 5 s. The dissociation curve method was applied, according to the manufacturer's protocol (60°C to 95°C), to ensure the presence of a single specific PCR product. The standard curve method was used for analysis, and results are expressed as *CXCL12*/*β-actin *ratios.

### Statistical analyses

For the series of 183 patients included in the GINECO clinical trial, the relationship between CXCL12 expression and clinical and pathologic features was assessed with *t*-tests (continuous variables) or Fisher's exact tests (binary variables). Overall survival (OS) was calculated from the date of inclusion to death and progression-free survival (PFS) was calculated from the date of inclusion until progression or last follow-up examination. Progression was defined as a 20% increase in the diameter of all measured lesions, the appearance of new lesions and/or the doubling of CA125 tumor marker concentration from baseline values. Kaplan-Meier analysis was carried out to generate PFS and OS curves. Univariate COX model analysis was carried out to assess the prognostic influence of clinical and biological variables. Hazard ratios (HR) and 95% confidence intervals (CIs) were determined. Analyses were performed with R software (The R Foundation for Statistical Computing ISBN 3-900051-07-0, http://www.r-project.org). For comparisons of *CXCL12 *mRNA levels in EOC samples, we used unpaired two-tailed Student's *t *tests (Prism software, GraphPad). *P *values <0.05 were considered statistically significant.

## Results

### Detection of CXCL12 products in healthy and malignant ovarian epithelial cells

**Figure 1:**
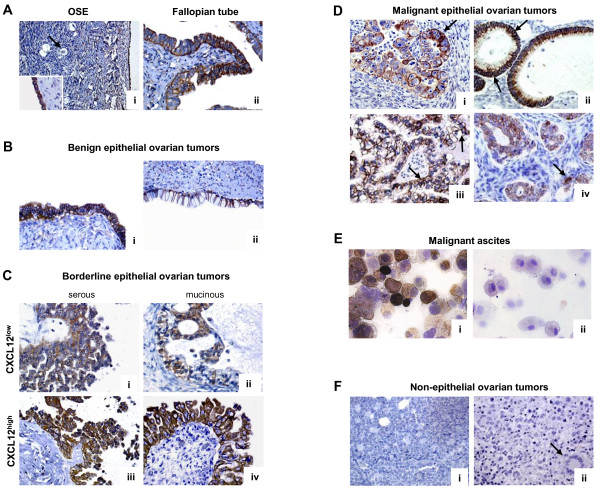
**CXCL12 expression in healthy and malignant ovaries**. **(A) **Healthy ovary (i), CXCL12 immunoreactivity in OSE (inset is the outlined region on the tissue specimen containing surface epithelium, × 40), faint staining in the stroma and no signal in follicles and oocytes (arrow) (× 20); fallopian tube (ii), CXCL12 immunoreactivity in cells of the epithelium (× 40). **(B) **Serous (i) and mucinous (ii) benign epithelial ovarian tumors, CXCL12 immunoreactivity in proliferating epithelial cells (× 40). **(C) **Serous (i and iii) and mucinous (ii and iv) borderline epithelial ovarian tumors with low (CXCL12^low^, i and ii) or high (CXCL12^high^, iii and iv) levels of CXCL12 staining (× 40). **(D) **Malignant epithelial ovarian tumors: serous (i), mucinous (ii), clear-cell (iii) and endometrioid (iv), CXCL12 immunoreactivity in epithelial cells is confined to the cytoplasm, with frequent strong staining of the membrane (arrows), no staining in the nuclei of tumor cells or in the stroma (× 40). **(E) **Cytocentrifuged CD326^+ ^epithelial (i) and CD326^- ^non epithelial (ii) cells isolated from malignant ascites collected from a patient diagnosed with invasive EOC, CXCL12 is detected only in CD326^+ ^cells (× 40). **(F) **Non epithelial ovarian tumors: granulosa tumor (i) and dysgerminoma with characteristic morphological features, *i.e. *Exner bodies (arrow) (ii), absence of CXCL12 immunostaining from both tumors (× 40). No labeling was detected when the K15C anti-CXCL12 mAb was omitted or a 100-fold molar excess of recombinant CXCL12 was added to the mAb before incubation with tissues.

**Figure 2:**
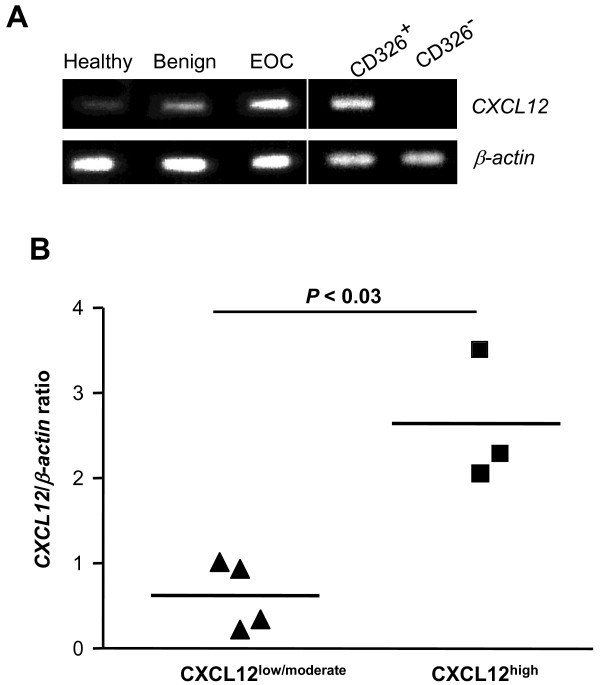
**Steady-state levels of *CXCL12 *transcripts in healthy and malignant ovarian tissues**. **(A) ***CXCL12 *mRNA was detected by conventional PCR and was of the expected size (417 bp) in healthy ovaries (faint signal), benign and invasive ovarian tumors (strong signal) and in CD326^+ ^epithelial cell-enriched malignant ascites samples. By contrast, *CXCL12 *transcripts were absent from CD326^- ^non epithelial cells. The results presented are from one experiment representative of three carried out. The white vertical line separates lanes not run on the same gel. **(B) ***CXCL12 *mRNA levels were quantified by real-time PCR and are expressed as *CXCL12*/*β-actin *ratios. The diagram shows the distribution of values and means for EOC samples identified as CXCL12^high ^and CXCL12^low/moderate^. Each symbol represents an individual sample run in duplicate. The *P *value presented is that for a two-tailed Student's *t *test.

The cellular expression of CXCL12 was examined by IHC on sections isolated from five healthy ovaries, eight serous or mucinous benign tumors (some still containing normal ovarian tissue), eight serous or mucinous borderline epithelial tumors, three non epithelial ovarian tumors (*i.e. *2 granulosa tumors and 1 dysgerminoma), and 183 invasive EOC. CXCL12 was clearly detected in cells of the OSE and the fallopian tube epithelium (Figure 1A). By contrast, CXCL12 was absent from both ovarian follicles and oocytes. CXCL12 immunoreactivity was detected in epithelium-derived proliferating tumor cells from benign tumors (Figure 1B). In both serous and mucinous borderline tumors and in serous, clear-cell, endometrioid and mucinous EOC specimens, CXCL12 was heterogeneously distributed in malignant cells, defining low and high expression profiles (Figures 1C and 1D). CXCL12 was confined to the cytoplasm of malignant epithelial cells, with particularly strong staining of the membrane frequently observed, and was not detected in nuclei (Figure 1D). CXCL12 was barely detectable in the stroma, and tumor epithelial cells are thus probably the principal source of CXCL12 in EOC. Epithelial cells isolated from malignant ascites and identified as CD326^+ ^cells were stained for CXCL12, whereas their CD326^- ^non epithelial counterparts, consisting mostly of CD45^+ ^leukocytes, were not stained for CXCL12 (Figure 1E). CXCL12 was also absent from non epithelial ovarian tumors (Figure 1F). A similar pattern was observed for CXCL12 mRNA, as shown by conventional and real-time PCR (Figure 2). CXCL12 was assayed in the culture medium of three ovarian cancer cell lines, SKOV-3, OVCAR-3 and BG-1, and in malignant ascites. We found that CXCL12 was produced in all cases, at concentrations of 2 to 10 ng/ml. Thus, the epithelial cells of both the OSE and fallopian tubes constitutively produce CXCL12 in the reproductive tracts of healthy women. CXCL12 was recovered from benign, borderline and malignant epithelial tumors but not from non epithelial ovarian tumors.

### Correlation of CXCL12 expression with clinical and pathological characteristics

We evaluated the prognostic value of CXCL12 for EOC, by quantifying CXCL12 staining in 183 ovarian cancer specimens. The mean age of the patients at initial diagnosis was 59 years (range 25-77); 68% of patients had serous adenocarcinomas and 85% had stage III/IV disease. Median follow-up for patients was 69 months. CXCL12 was heterogeneously distributed in tumor cells, with some cells displaying no detectable staining and others, strong immunoreactivity. CXCL12 expression ranged from high levels (scores 5-7) in 86 (47%) specimens to an absence of staining in 18 (<10%) cases. We applied a single cut-off at score 4, the median and mean value of the entire cohort, for the identification of samples producing low-moderate (CXCL12^low/moderate^, scores 0-4, n = 97) and high (CXCL12^high^, scores 5-7, n = 86) levels of CXCL12. The median age of the patients was 59 (range 25-77) in the CXCL12^low/moderate ^group and 57 (range 33-75) in the CXCL12^high ^group. There was thus no significant difference in patient age between these two groups (*P *= 0.31). Statistical analyses of CXCL12^high ^and CXCL12^low/moderate ^immunostaining and classical clinical parameters, such as histotype, HER2 status, FIGO stage, ascites and size of residual tumor after first laparotomy, revealed no significant correlation of CXCL12 status with any of the parameters tested (Table 1). Thus, CXCL12 expression does not reflect the clinical status of OEC.

**Table 1:** Correlation of CXCL12 expression with clinical parameters

	Patient number		***P *value**^**a**^
		
	**CXCL12**^**low/moderate**^	**CXCL12**^**high**^	
	Scores (0-4)	Scores (5-7)	
Histotype			
Serous (n = 125)	66	59	0.90 (NS)
Non serous (n = 58)	31	27	
HER2			
Negative (n = 171)	93	78	0.57 (NS)
Positive (n = 10)	4	6	
Undetermined (n = 2)			
FIGO stage			
I+II (n = 27)	14	13	0.91 (NS)
III+IV (n = 155)	82	73	
Undetermined (n = 1)			
Ascites			
Absence (n = 74)	37	37	0.45 (NS)
Presence (n = 86)	47	39	
Undetermined (n = 23)			
Residual tumor after initial laparatomy			
>1 cm (n = 83)	40	43	0.98 (NS)
≤1 cm (n = 24)	11	13	
Undetermined (n = 76)			

^a^Fisher's exact test. NS: not significant.

### Correlation of CXCL12 expression and patient outcome

**Figure 3:**
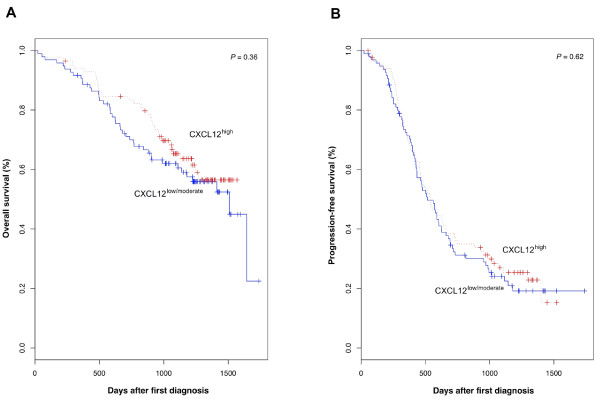
**Overall survival and progression-free survival as a function of CXCL12 expression**. Plots of Kaplan-Meier estimates for overall survival **(A) **and progression-free survival **(B) **of EOC patients with tumor tissues identified as CXCL12^low/moderate ^(n = 97, solid blue line) or CXCL12^high ^(n = 86, dotted red line). *P *values are those obtained in log-rank tests.

We then investigated whether CXCL12^high ^or CXCL12^low/moderate ^status affected OS and/or PFS. As expected, univariate analysis validated, for this series, known prognosis factors such as performance status, FIGO stage, presence of ascites and residual tumor after first laparotomy, which were associated with shorter OS and PFS (Table 2). In our large and homogeneous cohort, CXCL12 expression levels had no effect on OS or PFS (Table 2 and Figure 3). Thus, CXCL12 expression by tumor epithelial cells is not in itself a valuable prognostic factor in patients with advanced EOC.

**Table 2:** Hazard ratios for OS and PFS of 183 patients with EOC after univariate COX regression analysis of CXCL12 abundance and clinical and pathologic features

	Overall survival		Progression-free survival	
	**HR**^**a **^**[95% CI**^**b**^**]**	***P *value**	**HR [95% CI]**	***P *value**
		
CXCL12 (5-7) *vs *(0-4)	0.80 [0.51-1.28]	0.36	0.91 [0.65-1.29]	0.62
HER2 Positive *vs *negative	1.41 [0.68-2.90]	0.36	1.45 [0.84-2.51]	0.18
Age >60 *vs *≤60 years	1.40 [0.99-1.97]	<0.057	1.17 [0.90-1.51]	0.22
Performance status 1+2 *vs *0	1.72 [1.20-2.48]	<0.003	1.45 [1.11-1.88]	<0.005
FIGO stage III+IV *vs *I+II	5.55 [2.27-13.60]	<0.0002	4.43 [2.65-7.39]	<0.0001
Ascites Presence *vs *absence	3.33 [2.16-5.14]	<0.0001	2.29 [1.71-3.05]	<0.0001
Residual tumor after initial laparotomy >1 cm *vs *≤1 cm	1.72 [1.03-2.88]	<0.04	1.97 [1.36-2.87]	<0.0004

^a^Hazard ratio. ^b^95% confidence interval.

## Discussion

In this study, we demonstrate the previously unappreciated constitutive expression of CXCL12 by healthy ovarian epithelial cells and ovarian epithelial tumor cells, whether benign or malignant. CXCL12 was recovered from both the OSE and the epithelium of the fallopian tubes, both of which are considered possible origins of EOC. By contrast, it was not detected in follicles and oocytes or in malignant tumor cells arising from them, granulosa tumors or dysgerminomas. Furthermore, CXCL12 expression correlated neither with clinical parameters nor with HER2 status in specimens from 183 patients with advanced primary EOC enrolled in a multicenter clinical trial of first-line TCG-based chemotherapy (GINECO study). The intratumoral production of CXCL12 does not reflect the morphological heterogeneity of EOC and has no impact on PFS or OS after adjustment for established prognostic factors. Thus, CXCL12 is expressed by ovarian epithelial cells before tumorigenesis and does not constitute a valuable prognostic factor in EOC patients.

Recent studies on the origin and histogenesis of EOC have proposed that type I tumors, which are believed to include all major histotypes, originate from the OSE, whereas type II tumors, which are thought to consist almost exclusively of high-grade serous carcinomas, arise from the distal region of fallopian tubes [4,24,34]. Our findings clearly demonstrate that CXCL12 is constitutively produced by the epithelial cells of the OSE, whereas such expression was not previously suspected [11,16]. This apparent discrepancy may result from differences between our experimental protocol and those used in previous studies. For example, we used an anti-CXCL12 mAb rather than a polyclonal Ab and an additional microwave pretreatment for antigen retrieval, both of which would have increased the sensitivity of immunostaining. CXCL12 was also recovered from the epithelial cells of fallopian tubes, which were recently identified as a possible origin of high-grade serous EOC and which have a Müllerian duct-derived embryologic origin in common with the OSE [23,28,35]. By contrast, CXCL12 was undetectable in follicles, oocytes and their malignant non epithelial counterparts. Thus, CXCL12 is a chemokine constitutively produced by epithelial ovarian cells, from both healthy and malignant tissues. CXCL12 is present in ovarian epithelial cells before they become malignant and is therefore not useful as a marker of malignancy in EOC.

Scotton and coworkers reported a trend toward stronger CXCL12 expression in higher grade tumors [16]. However, Pils and coworkers recently found that the abundance of CXCL12 did not differ between borderline and malignant tumors [21]. In the present work, CXCL12 expression was detected in benign tumors as well as in borderline and malignant tumors. Although CXCL12 is unevenly distributed in low-grade (*i.e. *borderline and stage I) and in more advanced stage tumors, we have no evidence that its expression level is weaker in low-grade tumors. Among CXCL12-positive EOC specimens, we observed no significant differences in the fraction of CXCL12^high^-producing tumors for the four histotypes examined (*i.e. *serous, clear-cell, endometrioid and mucinous), despite previous reports of differences in epidemiologic and genetic changes, tumor markers and response to treatment (reviewed in [11]). We suggest that CXCL12 production levels overlap with EOC histotype differentiation and staging. Consistent with previous findings [16,22], CXCL12 was detected in more than 90% of patients with advanced primary EOC. However, it was barely detectable in the remaining cases (<10%), suggesting that *CXCL12 *expression might have been silenced, possibly through epigenetic mechanisms, such as promoter hypermethylation, a phenomenon already reported for colon carcinoma and breast cancer [36,37]. Indeed, further in-depth studies are required to determine whether transcriptional regulatory mechanisms account for heterogeneous CXCL12 production in EOC.

Recent studies have assessed the prognostic significance of CXCL12 expression in various cancers, including colorectal carcinoma [38], pancreatic ductal adenocarcinoma [39], breast cancer [40], esophageal squamous cell carcinoma [41], endometrial cancer [42], germ cell tumors [43] and EOC [21,22]. The study reported here was based on a large, homogeneous cohort of 183 patients, all given standard TCG-based chemotherapy. IHC showed that CXCL12 abundance was not correlated with any of the clinical parameters tested or with the HER2 status. Patients with CXCL12^high^-producing tumors had a PFS and OS similar to those of patients with CXCL12^low/moderate^-producing tumors. Consistent with the findings of smaller cohorts of patients given heterogeneous treatments [21,22], we therefore suggest that there is no evidence that CXCL12 production by malignant epithelial ovarian cells is of prognostic significance in EOC.

This lack of prognostic value for CXCL12 in EOC is somewhat puzzling, as this chemokine has been reported to enhance tumor cell proliferation and survival [16,18-20,44], to promote angiogenesis [17], to inhibit the host immune response [15] and to mediate resistance to hyperthermic intraperitoneal chemotherapy [45], which may favor tumor growth and spread. This apparent paradox may be explained by the cellular expression of CXCL12 not providing a true reflection of its bioavailability, which depends principally on the presence in the tumor microenvironment of factors capable of disrupting CXCL12 from glycosaminoglycans [46]. Moreover, CXCL12 activity may be mediated by two receptors, CXCR4 and CXCR7, and these receptors may also be rate-limiting elements. For many years, CXCL12 and CXCR4 were thought to act as an exclusive non redundant pair. However, the recent identification of RDC1/CXCR7 as a second receptor for CXCL12 has challenged this view, and we now need to determine the respective contributions of CXCR4 and CXCR7 to the homeostatic and pathological activities of CXCL12 [47-49]. The emerging possibility that CXCR7 acts as a decoy receptor provides further support for a potential role in EOC. Finally, the lack of influence of CXCL12 may reflect the unusual characteristics of metastases, predicting the occurrence of which is one of the major challenges in efforts to improve the clinical outcome of EOC. By contrast to breast cancer, in which distant metastases to the liver, lung and bone marrow are favored by high levels of CXCL12 expression in target organs and lower levels within the tumor [50], EOC spreads by the direct seeding of tumor cells into the peritoneal cavity, with preferential metastasis to local lymph nodes. In EOC, CXCL12 mRNA and protein have been detected mostly in the tumor cells themselves, and this feature has been reported for other cancers, including follicular lymphoma, pancreatic cancer, glioma and astrocytoma [10]. Ovarian epithelial tumor cells constitute a potent source of CXCL12. CXCL12 may therefore retain tumor cells at the site of production, rather than encouraging them to disseminate and to form secondary tumors in organs at some distance from the original tumor.

## Conclusions

Our findings highlight the previously unappreciated constitutive expression of CXCL12 by healthy epithelia of the ovary surface and the fallopian tubes, both these epithelia having been identified as probably sources of EOC. Thus, CXCL12 is expressed by epithelial cells before they become malignant. We also show that the level of CXCL12 expression in cancer cells is not a valuable prognostic factor in patients with advanced EOC. These findings do not exclude the possibility that CXCL12 contributes to tumor growth and spread via autocrine and/or paracrine action. There is therefore a need to determine whether CXCL12 status in EOC depends on its bioavailability and on the CXCR4/CXCR7 ratio in tumor cells, which would support an effect of CXCL12.

## List of abbreviations used

CI: Confidence interval; EOC: Epithelial ovarian cancer; FIGO: International Federation of Gynecology and Obstetrics; HR: Hazard ratio; IHC: Immunohistochemistry; OS: Overall survival; OSE: Ovarian surface epithelium; PFS: Progression-free survival; SDF-1: Stromal cell-derived factor-1; TCG: paclitaxel/carboplatin/gemcitabine; TMA: Tissue microarray; VEGF: vascular endothelium growth factor.

## Competing interests

The authors declare that they have no competing interests.

## Authors' contributions

Conceived and designed the experiments: VM, KB. Performed the experiments: FG, SN, LBD. Analyzed the data: VM, SCB, PB, SP, KB. Contributed reagents/materials/analysis tools: SCB, SP, EPL, JA, LG, FAS. Wrote the paper: VM, SCB, PB, KB. Provided funding: DE, KB. All authors read and approved the final manuscript.

## Pre-publication history

The pre-publication history for this paper can be accessed here:

http://www.biomedcentral.com/1471-2407/11/97/prepub

## References

[B1] Permuth-Wey J Sellers TA Epidemiology of ovarian cancer Methods Mol Biol 2009 472 413 437 full_text 19107446 10.1007/978-1-60327-492-0_20

[B2] Jemal A Siegel R Ward E Hao Y Xu J Murray T Thun MJ Cancer statistics, 2008 CA Cancer J Clin 2008 58 2 71 96 10.3322/CA.2007.0010 18287387

[B3] Shimizu Y Kamoi S Amada S Akiyama F Silverberg SG Toward the development of a universal grading system for ovarian epithelial carcinoma: testing of a proposed system in a series of 461 patients with uniform treatment and follow-up Cancer 1998 82 5 893 901 10.1002/(SICI)1097-0142(19980301)82:5<893::AID-CNCR14>3.0.CO;2-W 9486579

[B4] Shih Ie M Kurman RJ Ovarian tumorigenesis: a proposed model based on morphological and molecular genetic analysis Am J Pathol 2004 164 5 1511 1518 10.1016/S0002-9440(10)63708-X 15111296 PMC1615664

[B5] Zorn KK Bonome T Gangi L Chandramouli GV Awtrey CS Gardner GJ Barrett JC Boyd J Birrer MJ Gene expression profiles of serous, endometrioid, and clear cell subtypes of ovarian and endometrial cancer Clin Cancer Res 2005 11 18 6422 6430 10.1158/1078-0432.CCR-05-0508 16166416

[B6] Kobel M Kalloger SE Boyd N McKinney S Mehl E Palmer C Leung S Bowen NJ Ionescu DN Rajput A Ovarian carcinoma subtypes are different diseases: implications for biomarker studies PLoS Med 2008 5 12 e232 10.1371/journal.pmed.0050232 19053170 PMC2592352

[B7] Song H Ramus SJ Tyrer J Bolton KL Gentry-Maharaj A Wozniak E Anton-Culver H Chang-Claude J Cramer DW DiCioccio R A genome-wide association study identifies a new ovarian cancer susceptibility locus on 9p22.2 Nat Genet 2009 41 9 996 1000 10.1038/ng.424 19648919 PMC2844110

[B8] Schwartz DR Kardia SL Shedden KA Kuick R Michailidis G Taylor JM Misek DE Wu R Zhai Y Darrah DM Gene expression in ovarian cancer reflects both morphology and biological behavior, distinguishing clear cell from other poor-prognosis ovarian carcinomas Cancer Res 2002 62 16 4722 4729 12183431

[B9] White NM Mathews M Yousef GM Prizada A Popadiuk C Dore JJ KLK6 and KLK13 predict tumor recurrence in epithelial ovarian carcinoma Br J Cancer 2009 101 7 1107 1113 10.1038/sj.bjc.6605280 19707197 PMC2768090

[B10] Balkwill F Cancer and the chemokine network Nat Rev Cancer 2004 4 7 540 550 10.1038/nrc1388 15229479

[B11] Barbieri F Bajetto A Florio T Role of chemokine network in the development and progression of ovarian cancer: a potential novel pharmacological target J Oncol 2010 2010 426956 20049170 10.1155/2010/426956 PMC2798669

[B12] Lazennec G Richmond A Chemokines and chemokine receptors: new insights into cancer-related inflammation Trends Mol Med 2010 16 3 133 144 10.1016/j.molmed.2010.01.003 20163989 PMC2840699

[B13] Wilson J Balkwill F The role of cytokines in the epithelial cancer microenvironment Semin Cancer Biol 2002 12 2 113 120 10.1006/scbi.2001.0419 12027583

[B14] Kryczek I Wei S Keller E Liu R Zou W Stromal derived factor (SDF-1/CXCL12) and human tumor pathogenesis Am J Physiol Cell Physiol 2007 292 C987 95 10.1152/ajpcell.00406.2006 16943240

[B15] Zou W Machelon V Coulomb-L'Hermine A Borvak J Nome F Isaeva T Wei S Krzysiek R Durand-Gasselin I Gordon A Stromal-derived factor-1 in human tumors recruits and alters the function of plasmacytoid precursor dendritic cells Nat Med 2001 7 12 1339 1346 10.1038/nm1201-1339 11726975

[B16] Scotton CJ Wilson JL Scott K Stamp G Wilbanks GD Fricker S Bridger G Balkwill FR Multiple actions of the chemokine CXCL12 on epithelial tumor cells in human ovarian cancer Cancer Res 2002 62 20 5930 5938 12384559

[B17] Kryczek I Lange A Mottram P Alvarez X Cheng P Hogan M Moons L Wei S Zou L Machelon V CXCL12 and vascular endothelial growth factor synergistically induce neoangiogenesis in human ovarian cancers Cancer Res 2005 65 2 465 472 15695388

[B18] Scotton CJ Wilson JL Milliken D Stamp G Balkwill FR Epithelial cancer cell migration: a role for chemokine receptors? Cancer Res 2001 61 13 4961 4965 11431324

[B19] Kajiyama H Shibata K Terauchi M Ino K Nawa A Kikkawa F Involvement of SDF-1alpha/CXCR4 axis in the enhanced peritoneal metastasis of epithelial ovarian carcinoma Int J Cancer 2008 122 1 91 99 10.1002/ijc.23083 17893878

[B20] Barbolina MV Kim M Liu Y Shepard J Belmadani A Miller RJ Shea LD Stack MS Microenvironmental regulation of chemokine (C-X-C-motif) receptor 4 in ovarian carcinoma Mol Cancer Res 2010 8 5 653 664 10.1158/1541-7786.MCR-09-0463 20460402 PMC2883461

[B21] Pils D Pinter A Reibenwein J Alfanz A Horak P Schmid BC Hefler L Horvat R Reinthaller A Zeillinger R In ovarian cancer the prognostic influence of HER2/neu is not dependent on the CXCR4/SDF-1 signalling pathway Br J Cancer 2007 96 3 485 491 10.1038/sj.bjc.6603581 17245339 PMC2360022

[B22] Jiang YP Wu XH Shi B Wu WX Yin GR Expression of chemokine CXCL12 and its receptor CXCR4 in human epithelial ovarian cancer: an independent prognostic factor for tumor progression Gynecol Oncol 2006 103 1 226 233 10.1016/j.ygyno.2006.02.036 16631235

[B23] Auersperg N Wong AS Choi KC Kang SK Leung PC Ovarian surface epithelium: biology, endocrinology, and pathology Endocr Rev 2001 22 2 255 288 10.1210/er.22.2.255 11294827

[B24] Karst AM Drapkin R Ovarian cancer pathogenesis: a model in evolution J Oncol 2010 2010 932371 19746182 10.1155/2010/932371 PMC2739011

[B25] Camilleri-Broet S Hardy-Bessard AC Le Tourneau A Paraiso D Levrel O Leduc B Bain S Orfeuvre H Audouin J Pujade-Lauraine E HER-2 overexpression is an independent marker of poor prognosis of advanced primary ovarian carcinoma: a multicenter study of the GINECO group Ann Oncol 2004 15 1 104 112 10.1093/annonc/mdh021 14679128

[B26] Tuefferd M Couturier J Penault-Llorca F Vincent-Salomon A Broet P Guastalla JP Allouache D Combe M Weber B Pujade-Lauraine E HER2 status in ovarian carcinomas: a multicenter GINECO study of 320 patients PLoS One 2007 2 11 e1138 10.1371/journal.pone.0001138 17987122 PMC2042515

[B27] du Bois A Herrstedt J Hardy-Bessard AC Muller HH Harter P Kristensen G Joly F Huober J Avall-Lundqvist E Weber B Phase III trial of carboplatin plus paclitaxel with or without gemcitabine in first-line treatment of epithelial ovarian cancer J Clin Oncol 2010 28 4162 4169 10.1200/JCO.2009.27.4696 20733132

[B28] Drapkin R Crum CP Hecht JL Expression of candidate tumor markers in ovarian carcinoma and benign ovary: evidence for a link between epithelial phenotype and neoplasia Hum Pathol 2004 35 8 1014 1021 10.1016/j.humpath.2004.04.014 15297969

[B29] Redjimi N Gaudin F Touboul C Emilie D Pallardy M Biola-Vidamment A Fernandez H Prevot S Balabanian K Machelon V Identification of glucocorticoid-induced leucine zipper as a key regulator of tumor cell proliferation in epithelial ovarian cancer Mol Cancer 2009 8 83 10.1186/1476-4598-8-83 19814803 PMC2763858

[B30] Coulomb-L'Hermin A Amara A Schiff C Durand-Gasselin I Foussat A Delaunay T Chaouat G Capron F Ledee N Galanaud P Stromal cell-derived factor 1 (SDF-1) and antenatal human B cell lymphopoiesis: expression of SDF-1 by mesothelial cells and biliary ductal plate epithelial cells Proc Natl Acad Sci USA 1999 96 15 8585 8590 10411919 10.1073/pnas.96.15.8585 PMC17560

[B31] Balabanian K Couderc J Bouchet-Delbos L Amara A Berrebi D Foussat A Baleux F Portier A Durand-Gasselin I Coffman RL Role of the chemokine stromal cell-derived factor 1 in autoantibody production and nephritis in murine lupus J Immunol 2003 170 6 3392 3400 12626600 10.4049/jimmunol.170.6.3392

[B32] Kryczek I Frydman N Gaudin F Krzysiek R Fanchin R Emilie D Chouaib S Zou W Machelon V The chemokine SDF-1/CXCL12 contributes to T lymphocyte recruitment in human pre-ovulatory follicles and coordinates with lymphocytes to increase granulosa cell survival and embryo quality Am J Reprod Immunol 2005 54 5 270 283 10.1111/j.1600-0897.2005.00307.x 16212649

[B33] Orimo A Gupta PB Sgroi DC Arenzana-Seisdedos F Delaunay T Naeem R Carey VJ Richardson AL Weinberg RA Stromal fibroblasts present in invasive human breast carcinomas promote tumor growth and angiogenesis through elevated SDF-1/CXCL12 secretion Cell 2005 121 3 335 348 10.1016/j.cell.2005.02.034 15882617

[B34] Levanon K Ng V Piao HY Zhang Y Chang MC Roh MH Kindelberger DW Hirsch MS Crum CP Marto JA Primary *ex vivo *cultures of human fallopian tube epithelium as a model for serous ovarian carcinogenesis Oncogene 2010 29 8 1103 1113 10.1038/onc.2009.402 19935705 PMC2829112

[B35] Naora H Montell DJ Ovarian cancer metastasis: integrating insights from disparate model organisms Nat Rev Cancer 2005 5 5 355 366 10.1038/nrc1611 15864277

[B36] Wendt MK Johanesen PA Kang-Decker N Binion DG Shah V Dwinell MB Silencing of epithelial CXCL12 expression by DNA hypermethylation promotes colonic carcinoma metastasis Oncogene 2006 25 36 4986 4997 10.1038/sj.onc.1209505 16568088 PMC4610155

[B37] Zhou W Jiang Z Liu N Xu F Wen P Liu Y Zhong W Song X Chang X Zhang X Down-regulation of CXCL12 mRNA expression by promoter hypermethylation and its association with metastatic progression in human breast carcinomas J Cancer Res Clin Oncol 2009 135 1 91 102 10.1007/s00432-008-0435-x 18670789 PMC12160233

[B38] Akishima-Fukasawa Y Nakanishi Y Ino Y Moriya Y Kanai Y Hirohashi S Prognostic significance of CXCL12 expression in patients with colorectal carcinoma Am J Clin Pathol 2009 132 2 202 210 10.1309/AJCPK35VZJEWCUTL 19605814

[B39] Liang JJ Zhu S Bruggeman R Zaino R Evans D Fleming JB Gomez HF Zander DS Wang H High levels of expression of human stromal cell-derived factor-1 are associated with worse prognosis in patients with stage II pancreatic ductal adenocarcinoma Cancer Epidemiol Biomarkers Prev 2010 19 10 2598 2604 10.1158/1055-9965.EPI-10-0405 20732965

[B40] Hassan S Ferrario C Saragovi U Quenneville L Gaboury L Baccarelli A Salvucci O Basik M The influence of tumor-host interactions in the stromal cell-derived factor-1/CXCR4 ligand/receptor axis in determining metastatic risk in breast cancer Am J Pathol 2009 175 1 66 73 10.2353/ajpath.2009.080948 19497995 PMC2708795

[B41] Sasaki K Natsugoe S Ishigami S Matsumoto M Okumura H Setoyama T Uchikado Y Kita Y Tamotsu K Hanazono K Expression of CXCL12 and its receptor CXCR4 in esophageal squamous cell carcinoma Oncol Rep 2009 21 1 65 71 19082444

[B42] Gelmini S Mangoni M Castiglione F Beltrami C Pieralli A Andersson KL Fambrini M Taddei GL Serio M Orlando C The CXCR4/CXCL12 axis in endometrial cancer Clin Exp Metastasis 2009 26 3 261 268 10.1007/s10585-009-9240-4 19199057

[B43] Gilbert DC Chandler I McIntyre A Goddard NC Gabe R Huddart RA Shipley J Clinical and biological significance of CXCL12 and CXCR4 expression in adult testes and germ cell tumours of adults and adolescents J Pathol 2009 217 1 94 102 10.1002/path.2436 18839394

[B44] Barbero S Bonavia R Bajetto A Porcile C Pirani P Ravetti JL Zona GL Spaziante R Florio T Schettini G Stromal cell-derived factor 1 alpha stimulates human glioblastoma cell growth through the activation of both extracellular signal-regulated kinases 1/2 and Akt Cancer Res 2003 63 8 1969 1974 12702590

[B45] Lis R Touboul C Mirshahi P Ali F Mathew S Nolan DJ Maleki TM Abdalla SA Raynaud CM Querleu D Tumor associated mesenchymal stem cells protects ovarian cancer cells from hyperthermia through CXCL12 Int J Cancer 2010 128 715 725 10.1002/ijc.25619 20725999

[B46] Laguri C Arenzana-Seisdedos F Lortat-Jacob H Relationships between glycosaminoglycan and receptor binding sites in chemokines-the CXCL12 example Carbohydr Res 2008 343 12 2018 2023 10.1016/j.carres.2008.01.047 18334249

[B47] Balabanian K Lagane B Infantino S Chow KY Harriague J Moepps B Arenzana-Seisdedos F Thelen M Bachelerie F The chemokine SDF-1/CXCL12 binds to and signals through the orphan receptor RDC1 in T lymphocytes J Biol Chem 2005 280 42 35760 35766 10.1074/jbc.M508234200 16107333

[B48] Thelen M Thelen S CXCR7, CXCR4 and CXCL12: an eccentric trio ? J Neuroimmunol 2008 198 1-2 9 13 10.1016/j.jneuroim.2008.04.020 18533280

[B49] Burns JM Summers BC Wang Y Melikian A Berahovich R Miao Z Penfold ME Sunshine MJ Littman DR Kuo CJ A novel chemokine receptor for SDF-1 and I-TAC involved in cell survival, cell adhesion, and tumor development J Exp Med 2006 203 9 2201 2213 10.1084/jem.20052144 16940167 PMC2118398

[B50] Mirisola V Zuccarino A Bachmeier BE Sormani MP Falter J Nerlich A Pfeffer U CXCL12/SDF1 expression by breast cancers is an independent prognostic marker of disease-free and overall survival Eur J Cancer 2009 45 14 2579 2587 10.1016/j.ejca.2009.06.026 19646861

